# Testing for Alcohol Responsiveness in Familial Essential Tremor

**DOI:** 10.5334/tohm.923

**Published:** 2024-06-11

**Authors:** Cheryl S. J. Everlo, Marina A. J. Tijssen, A. M. Madelein Van Der Stouwe

**Affiliations:** 1 Department of Neurology, University Medical Center Groningen (UMCG), Groningen, the Netherlands; 2 Expertise Center Movement Disorders Groningen, University Medical Center Groningen (UMCG), Groningen, the Netherlands

**Keywords:** Essential tremor, familial, tremor, alcohol

## Abstract

**Background:**

Essential tremor (ET) is the most common movement disorder in adults and is considered to be highly heritable. A reduction of the tremor amplitude after alcohol consumption is reported in approximately half of the patients. In this study, we describe the alcohol response in our familial ET cohort by employing an alcohol responsivity test designed by Knudsen et al. outside its original research group for the first time.

**Methods:**

We recruited families with at least three trembling family members and confirmed ET diagnoses. During the in-hospital alcohol responsivity test, tremor was measured using Archimedes spirals before alcohol consumption (T0), one hour after alcohol intake (T1), and the next morning (T2). The spirals were rated by two independent raters using the Bain Findley scale. The average of these two scores was calculated as the Archimedes Spiral Rating (ASR) for each time point.

**Results:**

Twenty-four confirmed ET patients were included for analysis. The median ASR at T0 (5.0) and T2 (4.75) were significantly higher than the median ASR at T1 (3.25) (both p < 0.001). In 67% of patients, a difference in ASR between T0 and T1 (dASR) ≥ 2 pointed towards an improvement of tremor after consuming alcohol.

**Discussion:**

We confirmed that the alcohol responsiveness test of Knudsen et al. is useful in determining objective alcohol responsivity. We established a significantly reduced ASR after alcohol consumption in 67% of familial ET patients in our cohort. In the future, a larger population is needed to establish whether familial aggregation of alcohol responsivity occurs in essential tremor patients.

**Highlights:**

- The test designed by Knudsen et al. effectively established objective alcohol responsiveness outside its original research group.
- We found an objective alcohol response in 67% of our familial ET cohort.
- Subjective VAS scores were significantly lower after alcohol consumption.
- There was no correlation between the objective and subjective alcohol responsiveness.
- Familial aggregation of alcohol responsiveness in ET should be studied in a larger cohort.

## 1. Introduction

Tremor is defined as an involuntary, rhythmic, oscillatory movement of a body part. Essential tremor (ET) is the most common movement disorder in adults and is considered to be a highly heritable disorder [1]. Estimates for the proportion of ET patients with a positive family history range from 20% to 90% [2,3,4].

Approximately half the patients with ET report a positive effect of alcohol on their tremor [5,6,7]. A low blood level of alcohol does not affect the tremor frequency but reduces the amplitude by 50–70% and, therefore, alcohol sensitivity is supportive for ET diagnosis. Whether alcohol responsiveness aggregates in families with ET has only been studied once despite it being such a notable phenomenon [8]. In that study on hereditary essential tremor [9], the self-reported responsiveness was either consistently present or absent in 80% of the families, suggesting the presence of alcohol responsiveness in other family members, although they did not compare this proportion to that seen in sporadic cases.

Recently, Knudsen, Lorenz and Deuschl developed an alcohol responsiveness test to objectively and safely assess alcohol responsiveness either in the hospital or at home [10] and showed that the correlation between self-reported and objective alcohol responsiveness was limited [11]. This means that, when investigating alcohol responsiveness, it is crucial to combine subjective and objective measures, such as the test designed by Knudsen et al.

In this study, we report on both subjective, self-reported alcohol responsiveness and objective alcohol responsiveness as measured with the previously mentioned test in a cohort of familial ET patients for the first time. Our goals are 1) to employ the test for alcohol responsiveness outside the original Kiel group for the first time, 2) to describe the alcohol response of our cohort, and finally 3) to establish whether objectified responsivity correlates with self-perceived alcohol responsiveness.

## 2. Methods

### 2.1 Participants

Participants were recruited through the department of Neurology of the University Medical Center Groningen, the Isala Clinics in Zwolle, or by means of self-registration after online promotion of the study. Participants were enrolled between November 2020 and March 2022, as part of a larger study on familial ET where participating families had to consist of a proband, diagnosed with ET by a neurologist or general practitioner, and minimally two trembling relatives of at least two generations. Participants under the age of 18 were excluded, as were participants with a history of alcoholism or a weekly alcohol intake of more than 20 units, participants who were pregnant or breastfeeding, or participants using medication incompatible with alcohol intake. Whether participants reported their tremor to be responsive to alcohol or not was not relevant for inclusion. By unanimous consensus of the authors (CSJE, MAJT, AMMS), ET diagnoses were confirmed when they met the MDS criteria for ET established in 2018 [12]. The authors’ consensus was based on evaluation of the questionnaires (e.g. demographic features, tremor features, medical history, and medication) and videotaped neurological examinations, with a detailed assessment of postural, kinetic, intention, and rest tremor and of potential additional symptoms (dystonia, ataxia, parkinsonism). To assess tremor severity the FTM-TRS scale was used. The local medical ethics council accepted the study protocol (METc Groningen 2019/683), and insisted that the first part of the alcohol responsiveness test would be performed in-hospital rather than at home as was the case in the study by Hopfner and colleagues, because of safety concerns. All participants gave written informed consent.

### 2.2 The alcohol responsiveness test by Knudsen et al

In advance of the alcohol responsiveness test, participants completed a questionnaire that included the question whether they considered their tremor improved after alcohol intake.

On the day of testing, participants were asked not to eat four hours before alcohol intake. The test took place in the hospital; we used the same method that has been previously described and validated in ET patients [10,11]Participants consumed a prescribed amount of alcohol within 20 minutes, aimed to reach a blood alcohol concentration of 0.6‰ as calculated by the Widmark formula. This formula is commonly used to estimate the amount of alcohol needed to reach a certain blood alcohol concentration, as follows: amount of alcohol (gr) = weight (kg) × R × 0.6 (desired concentration), with R being a factor for body distribution: 0.7 for men, 0.6 for women [13]. The source of alcohol was beer (5%) or wine (12%) according to the participant’s preference. The number of millilitres to consume was calculated for each participant individually.

**Figure 1:**
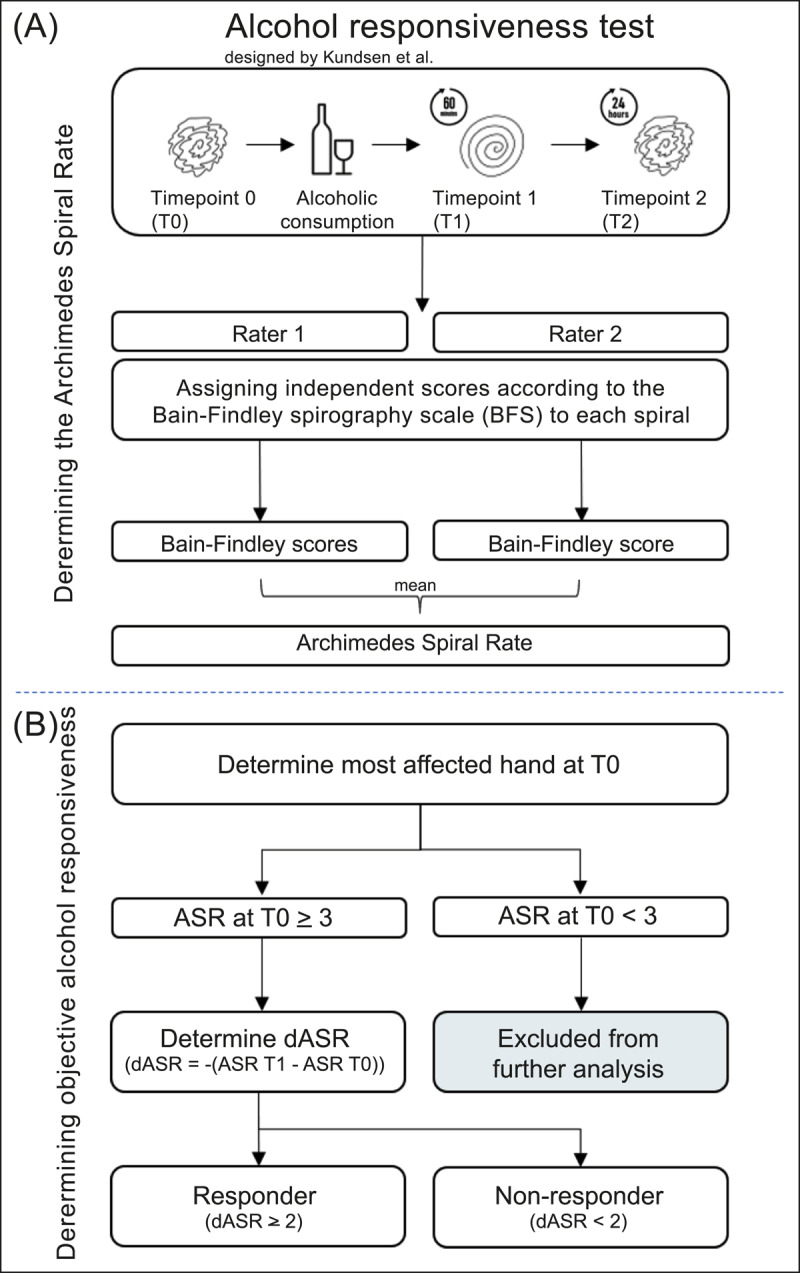
Flowchart of the alcohol responsiveness test designed by Knudsen et al. **A)** First, the Archimedes Spiral Ratings (ASR) are determined. Per participant, 6 spirals are scored independently by two raters, using the Bain-Findley spirography scale. Per spiral, the ASR is determined by calculating the mean of these Bain-Findley scores of both raters. **B)** Secondly, the objective alcohol responsiveness is determined for each participant. After determining the most affected hand at T0, an ASR of 3 or higher allows for calculation of the dASR and classification as either a responder (dASR ≥ 2) or non-responder (dASR < 2).

We assessed tremor severity at three different time points: before alcohol intake (T0), one hour after alcohol intake (T1), and the next morning (T2) to assess the occurrence of a so-called rebound effect (Figure 1a). For each assessment, two Archimedes spirals, a three-turn and a five-turn spiral, were drawn with each hand, starting at the middle and spiralling outwards, without supporting the arm. Additionally, participants rated their perceived tremor severity for both hands at each specific time point (T0, T1 and T2) using a visual analogue scale ranging from 1–100 (VAS), a recording of their overall subjective impression of tremor severity at that particular moment in time. Measurements at T0 and T1 were taken on-site, and spirals and VAS at T2 were drawn by the participants at home the next morning and sent in by post.

Two trained and blinded individuals (CSJE, AMMS) rated the Archimedes spirals semi-quantitatively according to the Bain-Findley spirography scale on a 10-point scale. On this scale, a score of zero represents an undetectable tremor, a score of nine indicates a severe tremor, and the maximum score of ten is given if a patient is unable to draw a spiral. Training was conducted by independently rating 16 spirals by both raters. These were selected by an independent researcher, who ensured these spirals represented a diverse range in tremor severity. After the independent assessment by both raters, they agreed upon discussion on scores that varied using the Bain-Findley spirography handbook (kindly provided as PDF by Dr. P.G. Bain) [14]. After this training session, all spirals were coded with a random index number and presented to the raters in a randomised sequence, thus blinding the raters to the identity of the participant as well as the time point (T0, T1 or T2). The two ratings were performed independently, and the average of the two ratings was calculated as the Archimedes spiral rating (ASR) for each time point (Figure 1a). Because the five-turn spiral resembles the original Bain Findley spirographs most closely and to minimise the floor effect that is associated with clinical rating scales including spirography, we decided to perform further calculations of alcohol responsiveness using only the five-turn spirals (Supplementary Material). Although ET is always bilateral, a slight asymmetry may exist, which is why we compared alcohol responsiveness between the most and the least affected hand (Supplementary Material). We used the ASR of the most affected hand at T = 0 for further calculations on objective alcohol responsiveness.

Next, we calculated the change in tremor severity after alcohol intake (Figure 1b). Given the sensitivity to change and a floor effect of the spiral ratings as established in previous studies [10,11,15], an initial ASR of at least 3 (mild tremor) is required to reliably establish the presence or absence of alcohol responsiveness. Therefore, all patients with an initial ASR below 3 (with negligible tremor) were excluded from further analysis. We calculated the difference in ASR (dASR) between T0 and T1 only in participants with an initial ASR at T0 of ≥ 3 points, because a dASR of ≥ 2 points is the cut-off above which alcohol responsiveness or lack thereof can reliably be established, as shown by Hopfner et al. [11].

### 2.3 Statistics

Statistical analysis was performed using SPSS 28.0. Descriptive statistics are presented as absolute numbers (percentages); mean, standard deviation and range; median, inter-quartile range (IQR) and range. Normality was verified by the Shapiro-Wilk test (< 50 samples) or the Kolmogorov-Smirnov test (≥50 samples). Because the Bain-Findley spirography scale is ordinal and there were two raters, the inter-rater reliability was determined using the intraclass correlation coefficient, which reflects consistency between raters (Supplementary Material). Differences in ASR at the different time points were determined by the Wilcoxon rank test (paired samples) or the Man Whitney U test (unpaired samples). The Spearman-Rho test was applied to assess linear correlations. We compared the proportion of alcohol responsive patients in our cohort and the cohort reported by Hopfner et al. using a Chi-square test. Significance was accepted at *p <* 0.05.

## 3. Results

### 3.1 Participants

Twenty-eight participants completed the study protocol, belonging to 9 families. Exclusions after reconfirming the diagnosis led to a final sample of 24 participants: eight probands and sixteen relatives (13 first-degree and 3 second-degree). Two of the four patients whose ET diagnosis was not confirmed had no tremor at T0, one presented with myoclonus, and one had a dystonic tremor. Detailed characteristics of these confirmed ET participants are shown in Table 1. The mean age at registration of all participants with confirmed ET was 58.2 years (SD 17.1, range 24–91), a median age of onset of 18 years (IQR 27, range 0–80), and a median total tremor score based on the FTM-TRS of 17 (IQR 21, range 3–46). Information on characteristics of individual examination results and probands vs relatives can be found in the Supplementary Material.

**Table 1:** Demographic and clinical characteristics.


	PARTICIPANTS WITH CONFIRMED ET DIAGNOSIS (n = 24)

Female gender	8 (33)

Age at registration (years)	58.2 ± 17.1, 24–91

Age of onset (years)	18, IQR 27, 0–80^a^

Disease duration^b^ (years)	21.5, IQR 39, 6–69

Right-handedness	20 (83)

Total tremor score^c,d^ (FTM-TRS)	17, IQR 21, 3–46

FTM-TRS part A	3.5, IQR 6, 0–18

FTM-TRS part B^d^	9, IQR 12, 2–26

FTM-TRS part C	6.2 ± 4.6, 0–14

Relationship to proband	

Self	8 (33)

First degree	13 (54)

Second degree	3 (13)

Currently taking medication for ET	7 (29)

Number of alcoholic drinks per week	5.5, IQR 7, 0–28

Positive self-reported alcohol responsiveness of their tremor	12 (50)


*All values are mean ± SD, range; median, IQR, range; or number (%)*. *NA, not applicable*. *^a^ One missing value.* *^b^ Calculated as follows: age at registration – age of onset*. *^c^ Maximum score total Fahn-Tolosa-Marin Tremor Rating Scale (FTM-TRS) = 92, part A = 24, part B = 36, part C = 32. If (partial) sub-scores were missing, patients were excluded from analysis (N = 17)*. *^d^ (Part of) FTM-TRS part B is missing in 7 patients, these were excluded from analysis (N = 17)*.

### 3.2 Results of the alcohol responsiveness test

Prior to analysis of objective alcohol responsiveness, we established that the interrater reliability between the two independent raters showed excellent agreement (ICC 0.938, more information can be found in the Supplementary Material).

**Figure 2:**
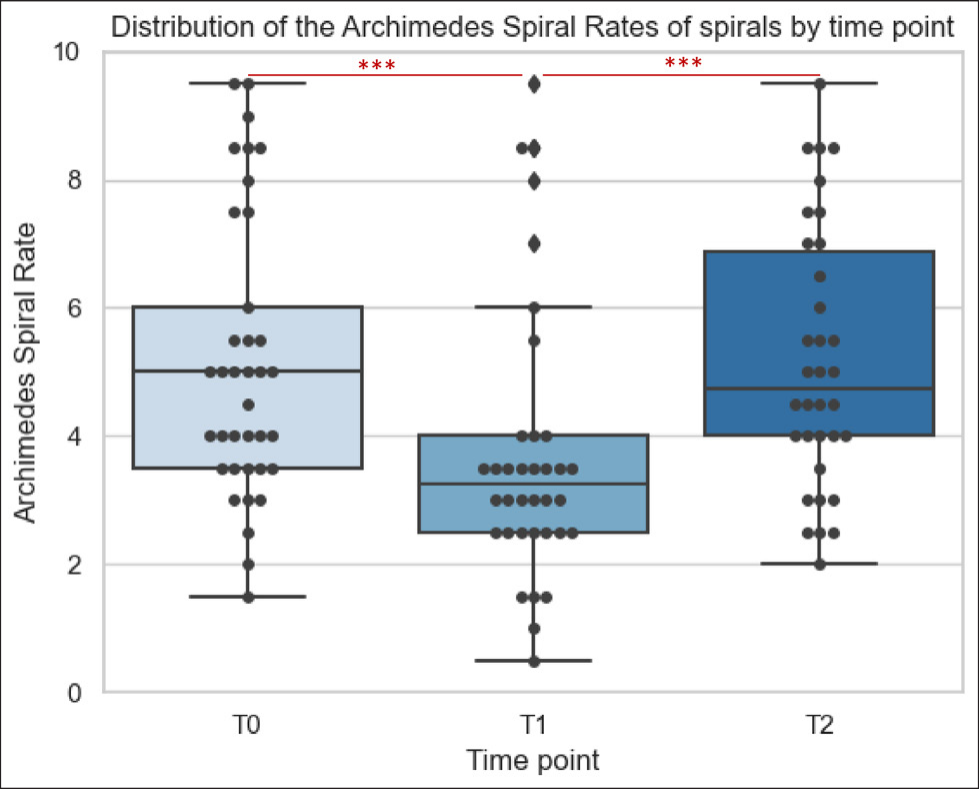
Distribution of the Archimedes Spiral Ratings (ASRs) of all reconfirmed ET participants for each time point (five-turn spirals, dominant and non-dominant hand). *N* = 107 spirals. Asterisks (***) indicate significant differences between the two conditions (*p* < 0.001). Diamonds indicate outliers in the data. *T0*: before alcohol intake; *T1*: one hour after alcohol intake; *T2*: morning after day of alcohol intake.

We display the distribution of the spirals at three different time points in Figure 2 to illustrate how the tremor severity of the confirmed essential tremor patients changed over time in response to alcohol consumption. The median ASR before alcohol consumption (T0) of 5.0 (IQR 3.6, 2.0–9.5) is significantly higher than the median ASR of 3.25 (IQR 1.5, 0.5–9.5) after alcohol consumption (T1) (*p <* 0.001). The next morning (T2) the ASR of 4.75 (IQR 3.1, 1.0–9.5) was also significantly higher than after alcohol consumption (*p* < .001). We found no statistical difference between ASR at T0 and T2, i.e. no rebound effect (*p* = 0.166) (Table 2).

**Table 2:** Tremor severity as indicated by the Archimedes Spiral Rate before and after alcohol intake.


	AVERAGE TWO RATERS	RATER 1	RATER 2

T0	5.0 (IQR 3.6, 2.0–9.5)	5.0 (IQR 2.8, 2.0–9.0)	5.0 (IQR 3.5, 2.0–10.0)

T1	3.25 (IQR 1.5, 0.5–9.5)*	3.0 (IQR 1.0, 0.0–9.0)*	3.0 (IQR 1.8, 1.0–10.0)*

T2	4.75 (IQR 3.1, 2.0–9.5) **	5.0 (IQR 3.0, 2.0–9.0) **	5.0 (IQR 3.3, 2.0–10.0) **


All values are displayed as median (interquartile range, range), with a possible range from zero to ten on the Bain Findley Spirography scale. Asterixis indicate statistical differences p < 0.05 between *T1 and T0, ** T2 and T1.

**Figure 3:**
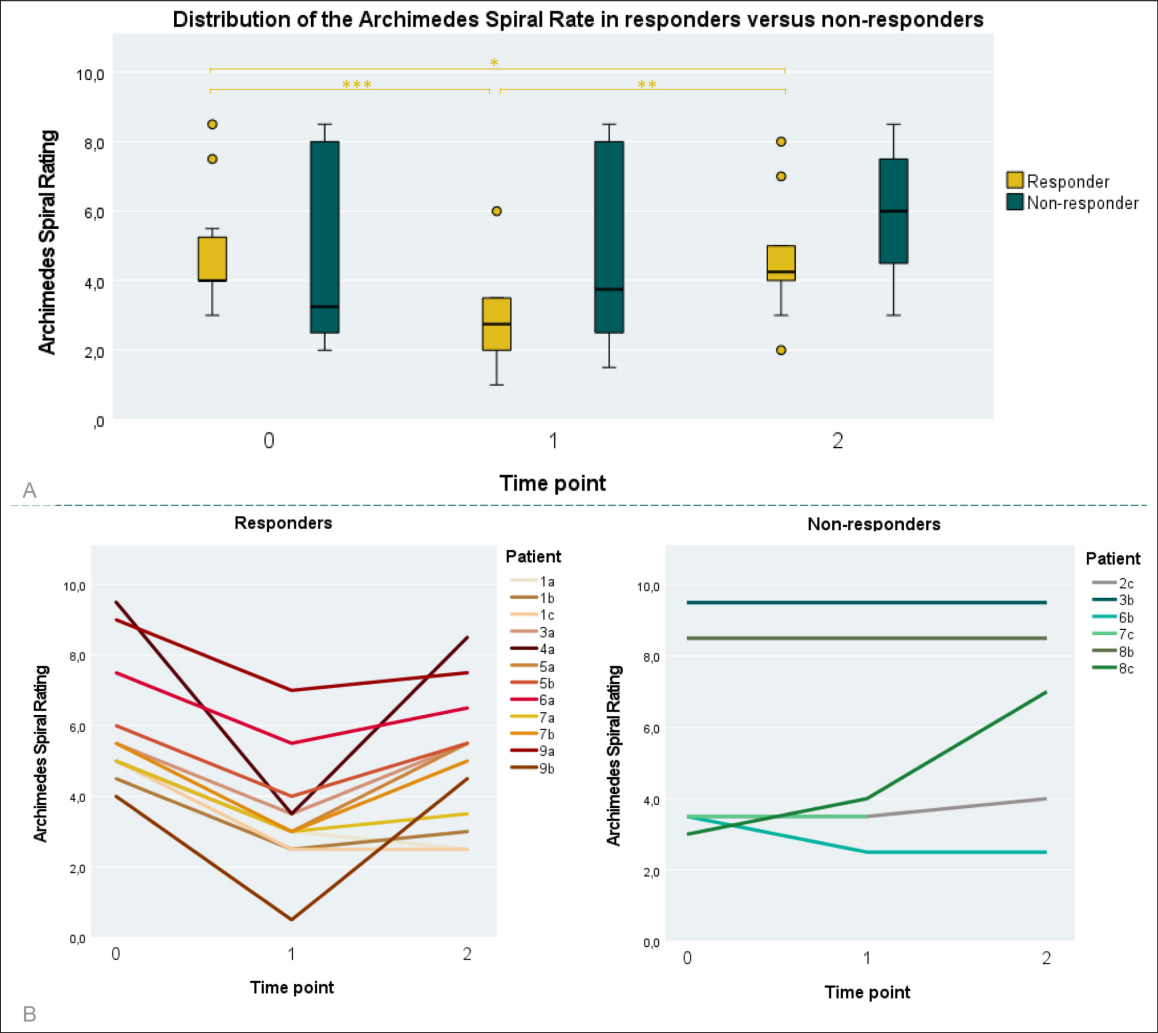
**A)** Distribution of the Archimedes Spiral Ratings (ASR) of responders versus non-responders for each time point (*N* = 53 spirals). There is no significant difference between the ASR of responders and non-responders at any time point. Responders’ median ASR at T0 is 5.25 (IQR 2.1; 4–9.5), at T1 3.0 (IQR 1.25; 0.5–7) and at T2 5.25 (IQR 3.13; 2.5–8.5). Non-responders’ median ASR at T0 3.5 (IQR 5.8; 3–9.5), at T1 4.0 (IQR 6.0; 2.5–9.5) and at T2 7.0 (IQR 5.75; 2.5–9.5). For the responders, significant differences were found between all time points. Asterisks (*) indicate significant differences between the two conditions (*p < 0.05, **p < 0.01, ***p < 0.001). *T0*: before alcohol intake; *T1*: one hour after alcohol intake; *T2*: morning after day of alcohol intake. **B**) Individual courses in time of the ASR split for responders and non-responders. Patient numbers indicate families, letters indicate individuals.

Eighteen out of 24 participants had an initial ASR ≥ 3 at T0 (75%), permitting objective determination of alcohol responsiveness with the alcohol responsiveness test (Figure 1b). In 12 of these participants (67%), the dASR ≥ 2 implies an improvement of their tremor after consuming alcohol in their most affected hand. The median dASR in alcohol-responsive patients was 2 (IQR 0.5, 2–6). There were 6 non-responders, in whom the median dASR was 0 (IQR 0.5, –1–1). Figure 3a shows the distribution of all ASRs among responders and non-responders per time point. For the responders, a significantly lower ASR on T2 compared to T0 (*p* < 0.05) implies the absence of a rebound effect. The individual courses of the ASRs for responders and non-responders are shown in Figure 3b. We found no statistical difference between the proportions of alcohol responsiveness in 12/18 patients (67%, 95% confidence interval for proportion 0.4528–0.8872) in our cohort versus in 48/104 patients (46%, 95% confidence interval for proportion 0.3642–0.5558) in the cohort reported by Hopfner et al (χ^2^ = 2.58, *p* = 0.108).

### 3.3 Subjective versus objective alcohol responsiveness

With regard to self-reported alcohol responsiveness, 50% of the participants indicated their tremor to be alcohol responsive before the test. When comparing subjective and objective measures, 66.7% of the objectively established alcohol responders believed their tremor to be alcohol responsive, while 33.3% of the non-responders did so as well. Statistically, we found no significant difference between responders and non-responders with regard to their own idea of the alcohol effect before the test (*p* = 0.358). When looking into the subjective VAS scores participants rated their tremor severity with during the experiment, we found that VAS scores were significantly lower after alcohol intake (median VAS at T0 = 50 (IQR 26, 18–73), at T1 = 29 (IQR 35, 12–70), *p* = 0.003) and back to normal the next morning (median VAS at T2 = 52 (IQR 17, 16–83), *p* = 0.917. We also studied the relationship between the dASR and the change in VAS before and after alcohol consumption (dVAS) and found no correlation between dASR and dVAS (*r^2^* = 0.263, *p* = 0.292).

### 3.4 Alcohol responsivity among families

**Figure 4:**
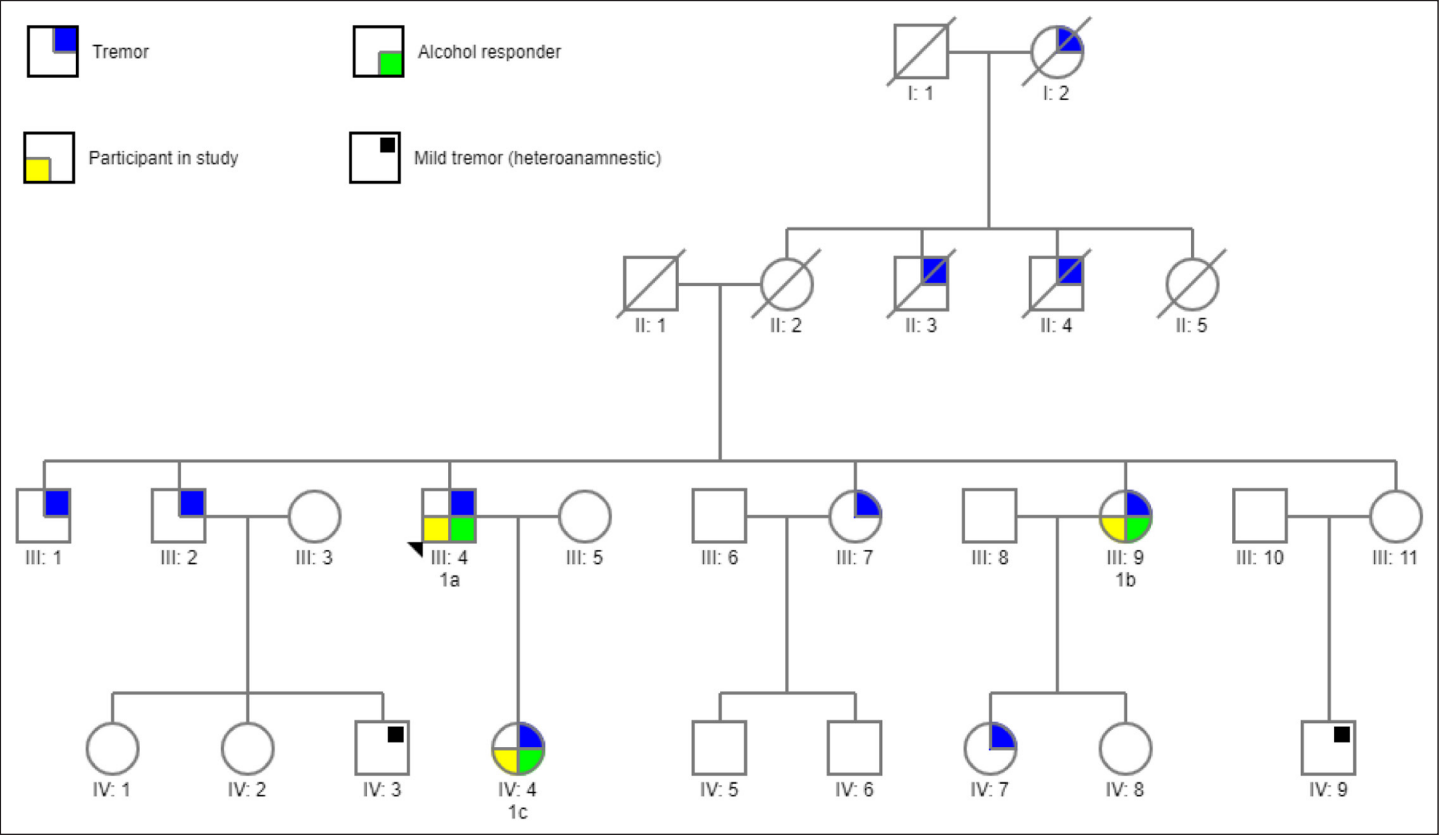
Pedigree of a family with alcohol-responsive essential tremor.

Despite the fact that there are only 9 families included in this study, we did take a preliminary look at the presence of tremor in other family members using the objective scores. In one family all three participants showed a dASR ≥ 2, implying this is an alcohol-responsive family (Figure 4). In addition, in three other families, objective alcohol responsiveness was found in two of the three participating family members. The other participating family member in these three families was 1) a non-responder in one case and 2) a person with mild tremor with ASR at T0 < 3 and therefore not analyzed further in the other two cases. Moreover, there was one family where two participants out of three had a dASR ≤ 2, indicating alcohol resistance. Among the four remaining families there was diversity in responders, non-responders or mildly affected participants.

## 4. Discussion

To the best of our knowledge, this is the first study describing a familial ET cohort in which alcohol responsiveness was objectively assessed, and where the validated alcohol responsiveness test by Knudsen et al. was used outside the original Kiel group where the test was developed.

In this cohort, we established alcohol responsiveness (dASR ≥ 2) in 67% of our patients. In a previous study by Hopfner et al., 46% was found to be alcohol responsive using the same test [11]. We could not establish a statistically significant difference between these proportions of alcohol responsive patients (*p* = 0.108), however, this may be explained by our small sample size and correspondingly high confidence interval. As such, it is imaginable that alcohol responsiveness may be more prevalent in familial than in sporadic ET. The cohort assessed by Hopfner and colleagues consisted of unrelated patients and a positive family history of ET was not part of the inclusion criteria [11].

Alcohol responsiveness has also been studied using accelerometery instead of spirography in the past [7] and more recently [16]. In these accelerometery studies, an alcohol response was found in respectively 60% and 80% in sporadic ET patients. Overall, this is the first study to report alcohol responsiveness using an *established* alcohol responsivity test in an exclusively *familial* cohort.

Contrary to Hopfner at al., we found no rebound effect the next morning, with ASR’s 4.25 before alcohol intake and 4.5 the day after. It is possible that a rebound effect occurred earlier (while patients were sleeping), and had already waned upon waking up the next day when the T2 spiral was drawn, because the interval between T0 and T2 typically exceeded 12 hours [17].

A limitation of this study we would like to address is the small sample size. While ET is a common movement disorder, we experienced difficulty with participant inclusion because the inclusion criterion of tremor in the proband and at least two relatives was hard to meet, and the strict evaluation and inclusion of only confirmed cases of ET meant that some participants were excluded. We felt it necessary to exclude four of the initial 28 cases, after clinical examination, review of the video’s and discussion among the authors, in accordance with the new diagnostic criteria [12]. These exclusions illustrate the known fact that ET is commonly misdiagnosed [18] and that what constitutes the syndrome of ET is subject of ongoing discussion [19,20].

Another reason for the small sample size relates to the alcohol responsiveness test designed by Knudsen et al., in which an initial ASR of at least 3 points is required to be able to investigate objective alcohol responsiveness. In other words: tremor needs to be severe enough to allow objective assessment of alcohol responsiveness. In this study, in 18 of the 24 patients the baseline tremor was sufficient for objective determination of the alcohol responsiveness, which means that 25% of our cohort was excluded from this analysis, a significant reduction of our sample size. As an aside, note that the fact that alcohol responsiveness can only be evaluated in mild-moderate-severe ET with this alcohol responsiveness test has implications for the investigation of families. As tremor severity is related to disease duration and age [21,22], younger family members are typically less affected, which means it may not be possible to assess alcohol responsiveness in the younger generations objectively. When investigating families, the implication is that only families with more severe tremor or families with only older relatives can be labelled as alcohol responsive.

In addition, the results invite us to speculate about the presence of alcohol responsivity in other family members. Familial aggregation of several clinical characteristics, such as age of onset, rate of progression, and tremor asymmetry have been studied before [8]. Alcohol responsiveness has only been investigated once before in a study on hereditary tremor [9]. In our study, we found that in one family, the tremor of all participating family members was objectively alcohol-responsive (Figure 4), which might suggest the presence of this feature in other family members. However, our small sample size does not allow us to draw conclusions on familial aggregation, for which an expansion of this familial ET cohort would be needed in the future, and ideally a non-ET control group. This could prove valuable, because alcohol responsiveness might be a feature indicative of a subgroup of familial ET, in which genetic testing might reveal a common pathogenic variant causing ET.

In conclusion, the alcohol responsiveness test was effective in establishing alcohol responsiveness objectively, although a limitation lies in the fact that tremor needs to be severe enough to allow assessment. We established a significantly reduced ASR after alcohol consumption in 67% of familial ET patients in our cohort. In the future, a larger population is needed to establish whether familial aggregation of alcohol responsiveness occurs in ET patients.

## Additional File

The additional file for this article can be found as follows:

10.5334/tohm.923.s1 Supplementary Material. Demographic and clinical characteristics of probands versus relatives; interrater reliability; detailed analysis of all Archimedes Spiral Rates.
