# Supplementary material for: Testing for Alcohol Responsiveness in Familial Essential Tremor
Source: Tremor Other Hyperkinet Mov (N Y). 2024 Jun 11;14:30. doi: 10.5334/tohm.923 (PMC11177841; doi:10.5334/tohm.923)
Supplement: Supplementary Material. — Demographic and clinical characteristics of probands versus relatives; interrater reliability; detailed analysis of all Archimedes Spiral Rates. [file tohm-14-1-923-s1.pdf]

Supplementary Material

Demographic and clinical characteristics of probands versus relatives

Probands did not differ from their relatives in terms of gender, age of onset, handedness, or whether they used medication for their tremor. However, relatives had a significantly lower age at registration than probands (p=0.04). Regarding tremor severity, a difference between probands' median score of 19, IQR 16 versus relatives' median score of 15, IQR 18 trended towards significance (p=0.12), with relatives scoring lower on part A (clinical assessment of tremor severity based on observation of tremor amplitude during rest, posture, movement and finger-to-nose manoeuvres, p=0.03) and part C (questionnaire on tremor-related impairments in daily life, p=0.04).

Participant	Gender	Age at registration (years)	Age of onset[a] (years)	Disease duration (years)	Confirmed ET	Handedness	Total tremor score[b]	FTM-TRS part A	FTM-TRS part B	FTM-TRS part C	Currently taking medication for ET	Number of alcoholic drinks per week	Positive self-reported alcohol responsiveness of their tremor
1a	M	73	14	59	Y	R	MV	4	MV	11	N	28	Y
1b	F	69	14	55	Y	R	MV	8	MV	6	N	7	Y
1c	F	27	15	12	Y	R	MV	4	MV	1	N	8	Y
2a	M	79	16	63	Y	R	17	3	9	5	N	2	N
2b	F	48	18	30	Y	L	5	1	2	2	N	6	Y
2c	M	51	37	14	Y	L	16	3	8	5	N	3	Y
3a	M	35	15	20	Y	R	17	2	12	3	N	4	Y
3b	M	69	0	69	Y	R	46	7	26	13	Y	14	Y
3c	F	29	16	13	N	L	25	5	13	7	N	0	Y
4a	M	82	71	11	Y	R	MV	18	MV	14	Y	21	Y
4b	F	54	48	6	Y	R	3	0	2	1	N	4	N
4c	F	52	47	5	N	R	2	0	2	0	N	10	N
5a	M	64	18	46	Y	R	28	6	13	9	Y	3	N
5b	F	91	80	11	Y	R	34	7	15	12	N	0	N
5c	F	37	16	21	N	L	6	0	4	2	N	3	Y
6a	M	77	67	10	Y	L	15	8	2	8	Y	6	Y
6b	M	53	28	25	Y	R	MV	3	MV	0	N	12	N
6c	M	24	13	11	Y	R	MV	2	MV	0	N	10	Y
7a	F	62	41	21	Y	R	13	8	3	10	N	3	N
7b	M	64	54	10	Y	R	23	9	10	4	N	0	N
7c	F	56	-	56	Y	R	17	2	8	7	N	1	N
8a	F	65	25	40	N	R	35	8	18	9	Y	2	N
8b	M	60	9	51	Y	R	41	6	22	13	Y	5	N
8c	M	37	15	22	Y	R	17	2	11	4	N	10	N
8d	F	53	10	43	Y	R	7	0	5	2	N	2	N
9a	M	72	36	36	Y	R	45	9	23	13	Y	21	Y
9b	M	47	31	16	Y	R	MV	3	MV	3	Y	10	Y
9c	M	49	28	21	Y	L	6	2	2	2	N	5	N

F, female; M, male; Y, yes; N, no; R, right; L, left; MV, missing value
[a] *Calculated as follows: age at registration – age of onset*
[b] *Maximum score total FTM-TRS = 92, part A = 24, part B = 36, part C = 32*

Supplementary Table 1. Individual examination results

Supplementary Table 2. Demographic and clinical characteristics of probands versus relatives

	**Probands** (n = 8)	**Relatives** (n = 16)	**Difference between probands and relatives**
Female gender	1 (13)	7 (44)	p = 0.19[a]
Age at registration (years)	68.0 ± 15.0, 25 – 82	53.3 ± 16.3, 24 – 91	***p* = 0.04**[b]
Age of onset (years)	27, IQR 45, 14 – 71	18, IQR 24, 0 – 80 [c]	p = 0.32[d]
Disease duration[e] (years)	28.5, IQR 43, 10 – 63	21.5, IQR 38, 6 – 69	p = 0.36[d]
Right-handedness	7 (88)	13 (81)	p = 1.00[a]
Total tremor score[f,g] (FTM-TRS)	19, IQR 16, 13 – 43	15, IQR 18, 0 – 46	p = 0.12[d]
FTM-TRS part A	7, IQR 6, 2 – 18	2.5, IQR 7, 0 – 9	***p* = 0.03**[d]
FTM-TRS part B[g]	9, IQR 10, 2 – 25	8, IQR 9, 0 – 26	p = 0.46[d]
FTM-TRS part C	8.6 ± 3.4, 3 - 14	4.7 ± 4.4, 0 - 13	***p* = 0.04**[b]
Relationship to proband			NA
Self	8 (100)	0 (0)	
First degree	0 (0)	13 (81)	
Second degree	0 (0)	3 (19)	
Currently taking medication for ET	4 (50)	3 (19)	p = 0.17[a]
Number of alcoholic drinks per week	5, IQR 18, 2 – 28	5.5, IQR 8, 0 – 14	p = 0.54[d]
Positive self-reported alcohol responsiveness of their tremor	5 (63)	7 (44)	p = 0.67[a]

All values are mean ± SD, range; median, IQR, range; or number (%).
NA, not applicable.
[a] *Fisher's exact test*
[b] *Independent t-test*
[c] *One missing value*
[d] *Mann-Whitney U test*
[e] *Calculated as follows: age at registration – age of onset*
[f] *Maximum score total FTM-TRS = 92, part A = 24, part B = 36, part C = 32*
[g] *Partially missing values in 6 patients*

Interrater reliability

All patients drew four spirals at each time point and because two patients did not send in their T2 spirals, overall, 328 spirals remained. For each spiral, the mean BFS of the two independent raters was taken as the ASR outcome. We assessed the inter-rater reliability between rater 1 (CSJE) and rater 2 (AMMS) using the intraclass correlation coefficient (ICC), which reflects the consistency between the raters. An ICC of 0.938, using a consistency definition, showed excellent agreement. In 46,8% (153 spirals) complete agreement was arranged. The distribution of the absolute differences between rater 1 and rater 2, shown in Supplementary Figure 1, demonstrates that 94,8% of all differences were ≤ 2 BFS and 99,7% ≤ 3 BFS. Therefore, a difference of more than 2 BFS can be detected with > 95% certainty.

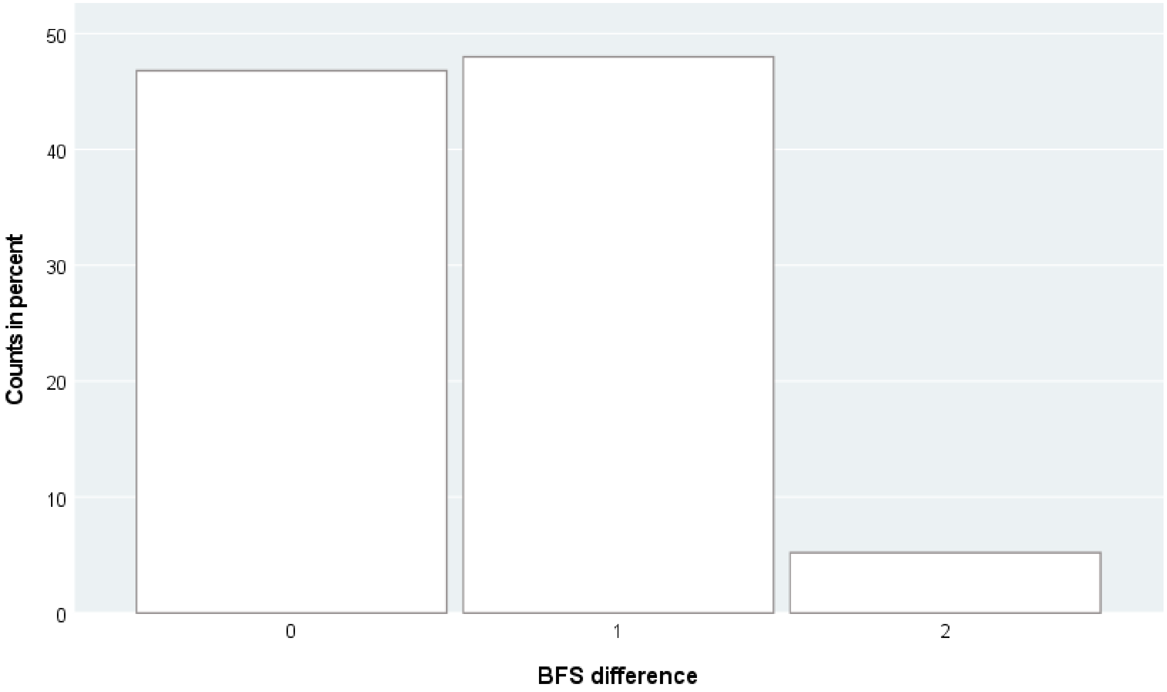

Supplementary Figure 1. Histogram of the percentage of the absolute differences between BFSs between both raters. N=327 differences were taken into account.

Detailed analysis of all Archimedes Spiral Rates

In paragraph 3.2 the changes in tremor severity within the five-turn spirals were described. In the three-turn spirals, these changes at the different time points persisted (T0 versus T1 and T1 versus T2: $p<0.001$). ASR was equal to or higher for the five-turn spiral than for the three-turn spiral at all time points (median 5.25 for three-turn vs 5.25 for five-turn at T0; 3.25 vs 3.5 at T1; 5.5 vs 5.5 at T2, all $p<0.001$). The ASRs of the three- and five-turn spiral were found to be highly correlated ($r^2 = 0.887$, $p<0.001$).

The changes at the different time points were maintained also when analysing all spirals, three-turn and five-turn from both the dominant and non-dominant hand (Supplementary Figure 2). The median ASR before alcohol consumption (T0) of 5.25 (IQR 4.4, 3-9.5) is significantly higher than the median ASR of 3.25 (IQR 3.3, 1.0-8.0) after alcohol consumption (T1) ($p<0.001$). The next morning (T2) the ASR of 5.5 (IQR 4.3, 3.0-8.5) was also significantly higher than after alcohol consumption ($p<0.001$). We found no statistical difference between ASR at T0 and T2, i.e. no rebound effect ($p=0.504$).

We examined the alcohol response between the most and least affected hands because, although ET is invariably bilateral, a little asymmetry may exist. At baseline (T0), the difference between the most and least affected hand was 0.75 ASR points ($p<0.001$). The decrease in tremor severity between T0 and T1 was significant in both hands ($p<0.001$, $p=0.004$), as was the increase in tremor severity between T1 and T2 ($p=0.001$, $p<0.001$).

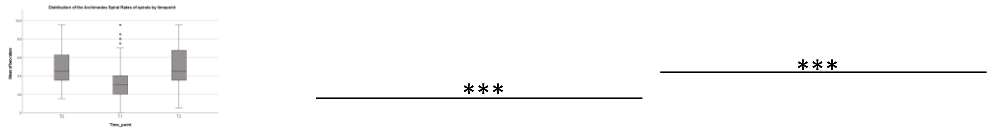

Supplementary Figure 2. Distribution of the Archimedes Spiral (ASRs) of all confirmed ET participants for each time point (five-turn spirals and three-turn spirals, dominant and non-dominant hand). *N*=212 spirals. Asterisks (***) indicate significant differences between the two conditions (p<0.001).
T0: before alcohol intake; *T1*: one hour after alcohol intake; *T2*: morning after day of alcohol intake.
